# Iliopsoas and Gluteal Muscles Are Asymmetric in Tennis Players but Not in Soccer Players

**DOI:** 10.1371/journal.pone.0022858

**Published:** 2011-07-29

**Authors:** Joaquin Sanchis-Moysi, Fernando Idoate, Mikel Izquierdo, Jose A. L. Calbet, Cecilia Dorado

**Affiliations:** 1 Physical Education Department, Las Palmas de Gran Canaria University, Las Palmas de Gran Canaria, Spain; 2 Radiology Department, Clínica San Miguel, Pamplona, Spain; 3 Health Sciences Department, Public University of Navarre, Pamplona, Spain; University of Illinois at Champaign-Urbana, United States of America

## Abstract

**Purpose:**

To determine the volume and degree of asymmetry of *iliopsoas* (IL) and *gluteal muscles* (GL) in tennis and soccer players.

**Methods:**

IL and GL volumes were determined using magnetic resonance imaging (MRI) in male professional tennis (TP) and soccer players (SP), and in non-active control subjects (CG) (n = 8, 15 and 6, respectively).

**Results:**

The dominant and non-dominant IL were hypertrophied in TP (24 and 36%, respectively, P<0.05) and SP (32 and 35%, respectively, P<0.05). In TP the asymmetric hypertrophy of IL (13% greater volume in the non-dominant than in the dominant IL, P<0.01) reversed the side-to-side relationship observed in CG (4% greater volume in the dominant than in the contralateral IL, P<0.01), whilst soccer players had similar volumes in both sides (P = 0.87). The degree of side-to-side asymmetry decreased linearly from the first lumbar disc to the pubic symphysis in TP (r = −0.97, P<0.001), SP (r = −0.85, P<0.01) and CG (r = −0.76, P<0.05). The slope of the relationship was lower in SP due to a greater hypertrophy of the proximal segments of the dominant IL. Soccer and CG had similar GL volumes in both sides (P = 0.11 and P = 0.19, for the dominant and contralateral GL, respectively). GL was asymmetrically hypertrophied in TP. The non-dominant GL volume was 20% greater in TP than in CG (P<0.05), whilst TP and CG had similar dominant GL volumes (P = 0.14).

**Conclusions:**

Tennis elicits an asymmetric hypertrophy of IL and reverses the normal dominant-to-non-dominant balance observed in non-active controls, while soccer is associated to a symmetric hypertrophy of IL. *Gluteal muscles* are asymmetrically hypertrophied in TP, while SP display a similar size to that observed in controls. It remains to be determined whether the different patterns of IL and GL hypertrophy may influence the risk of injury.

## Introduction

*Iliopsoas* (IL) and *gluteal muscles* (GL) are antagonist muscle groups which play an important role in several athletic tasks. A predominant hypertrophy of *iliopsoas* provides an advantage for achieving a better performance during high speed running [1]. *Gluteal muscles* contribute to stabilize the pelvis during the frequent side-step cutting maneuvers performed in many sports [2]. Tennis and soccer are asymmetric sports which demand repeated unilateral actions. In consequence, several muscles are hypertrophied asymmetrically [3], [4]. It remains to be determined whether soccer and tennis are associated to asymmetrical hypertrophy of *iliopsoas* and *gluteal muscles*. This information could help to design more specific strength training programs and to prevent common overload injuries associated to *iliopsoas* and *gluteal muscles* in tennis and soccer players, i.e. chronic groin pain or low back pain [5], [6].

The psoas and iliacus muscles originate from the lumbar spine and iliac fossa, respectively, converge to become the *iliopsoas muscle* and insert onto the lesser trochanter of the femur as the *iliopsoas* tendon [7]. The main function of *iliopsoas muscle* is to flex the thigh on the pelvis and laterally flex the lower vertebral column, but also functions as a lateral hip rotator, contributes to maintain the erect position and assist in raising the trunk when the body is in a recumbent position [8], [9]. On the other hand, the *gluteal muscles* are *gluteus minimus*, *medius* and *maximus*. *Gluteus minimus* and *medium* arises from the outer surface of the ilium and inserts onto the greater trochanter, and *gluteus maximus* forms the prominence of the buttock and covers the ischial tuberosity and much of the *gluteus medius* [10]. The main functions of *gluteal muscles* are to extend, abduct and rotate the hip. *Gluteal muscles* are also fundamental in keeping the trunk in an upright position when the contralateral foot is raised and in stabilizing the knee joint when the leg extensors are relaxed [10], [11].

Soccer and tennis are asymmetric sports which demand the participation of IL and GL muscles in several actions. A study using electromyography showed that the *iliopsoas* of the dominant leg (the preferred leg to kick the ball) was the most active muscle during the entire kicking motion whilst *gluteus maximus* was moderately active during the acceleration phase of the kicking leg and increased its activity just before ball impact [12]. *Iliopsoas* and *gluteal muscles* are also very demanded in tennis [13], [14]. Studies using cinematography have shown that the players profit the linear momentum from the extension of the lower extremities to asymmetrically activate lower trunk muscles to produce power during tennis strokes [15]–[17]. This pattern of activation induce the asymmetric hypertrophy of trunk and arm muscles in professional [4], [18], [19] and in young tennis players [20], [21]. The asymmetric hypertrophy of IL and GL could increase the risk of common injuries associated to soccer and tennis, i.e. chronic groin pain [6], low back pain [5] or anterior cruciate ligament injuries [22]. However, it remains to be determined whether soccer and tennis players display asymmetrically hypertrophied IL and GL.

The main aim of this study was to determine the pattern and degree of hypertrophy of *iliopsoas* and *gluteal muscles* in professional soccer and tennis players, using non-active controls as a reference. A secondary aim was to determine if soccer and tennis induces an asymmetric hypertrophy of *iliopsoas* and *gluteal muscles*.

The hypothesis to be tested is that professional soccer is associated with an asymmetric development of *iliopsoas* and *gluteal muscles*, with greater volume in the dominant compared to the non-dominant side, reflecting greater stretch-shortening loads on the dominant leg during kicking; and that tennis is associated with a greater hypertrophy of the non-dominant *iliopsoas* and *gluteal muscles* to provide a solid foundation for the torques generated by the dominant arm during tennis strokes.

## Methods

### Subjects

Fifteen male professional soccer players (SP) from a first division team of the Spanish Football League, 8 male professional tennis players (TP) from the International Tennis Federation tour (Futures and Challengers tournaments) and 6 non-active men (control group: CG) agreed to participate in the study (Table 1). Participants of the CG had never been involved in regular physical exercise. The current dedication to sport specific training sessions and competitions was 25±6.7 h/week and 9 h/week for TP and SP, respectively. All subjects were informed about the potential benefits and risks of the study and gave a written consent to participate. The study was approved by the ethical committee of the University of Las Palmas de Gran Canaria. All soccer and tennis players started their sport practice before 12 years old. In thirteen SP the dominant leg was the right leg, whilst 2 subjects had left leg dominance. Six TP were right handed and two of them used the two hands backhand stroke. The two left handed players used a one hand backhand stroke. All controls were right handed. In TP, SP and CG participants, leg and arm dominance was in the same side except in 1 right handed tennis player who had the left leg as dominant and 1 left handed tennis player who had the right leg as dominant. For comparative purposes, in this article the dominant side of *iliopsoas* and *gluteal muscles* corresponds to the same side of the dominant arm.

**Table 1:** Physical characteristics of soccer players and control group and total and regional length of *iliopsoas* and *gluteal muscles* from L1/L2 to the pubic simphysis and (mean ± SD).

Variables	Tennis Players			Soccer Players			Controls		
Age (years)	21.9	±	3.8	26.2	±	5.2	27.5	±	8.1
Height (cm)	182.5	±	3.9	182.3	±	5.6	177.7	±	2.6 a
Body mass (Kg)	75.4	±	6.9	78.0	±	6.8	75.5	±	11.1
BMI	22.6	±	1.5	23.5	±	1.7	23.9	±	3.5
*Ilopsoas* length									
1^st^ segment	3.0	±	0.0	3.0	±	0.0	2.8	±	0.4
2^nd^ segment	3.4	±	0.5	3.3	±	0.5	3.2	±	0.4
3^rd^ segment	3.3	±	0.5	3.5	±	0.5	3.0	±	0.0^ c^
4^th^ segment	3.9	±	0.4	3.9	±	0.3	3.3	±	0.5^ b, d^
5^th^ segment	3.0	±	0.0	3.0	±	0.0	3.0	±	0.0
6^th^ segment	3.6	±	0.5	3.9	±	0.4	3.3	±	0.5^ b^
7^th^ segment	3.4	±	0.5	3.5	±	0.5	3.0	±	0.0^ c^
8^th^ segment	3.9	±	0.4	3.9	±	0.3	3.7	±	0.5
Total	27.4	±	2.0	28.1	±	1.6	25.5	±	1.8^ b^
*Gluteal muscles* length									
1^st^ segment	3.6	±	0.5	3.9	±	0.3	3.2	±	0.4^ c^
2^nd^ segment	4.1	±	0.4	4.5	±	0.5	3.7	±	0.5 ^e^
3^rd^ segment	4.0	±	0.0	4.1	±	0.3	3.3	±	0.5^ b, f^
4^th^ segment	4.4	±	0.5	4.9	±	0.3^ g^	4.0	±	0.0^ c^
Total	16.1	±	1.2	17.4	±	0.9^ h^	14.2	±	1.2^ c, f^

a P<0.05 CG vs. SP and CG vs. TP, ^b^ P<0.05 CG vs. SP, ^c^ P<0.001 CG vs. SP, ^d^ P<0.05 CG vs. TP, ^e^ P<0.01 CG vs. SP, ^f^ P<0.01 CG vs. TP, ^g^ P<0.05 TP vs. SP, ^h^ P<0.01 TP vs. SP

### Magnetic resonance imaging

Magnetic resonance imaging was used to determine the muscle CSA and muscle volume of the left and right *iliopsoas* and *gluteal muscles*. A 1.5 T MRI scanner (Philips Achieva 1.5 Tesla system, Philips Healthcare, Best, the Netherlands) was used to acquire 10-mm axial contiguous slices from trunk, abdomen and pelvis, without interslice separation. Sagittal, coronal and transverse localizers of the body were obtained to determine precisely the anatomic sites for image acquisition. Transverse MRI images at rest (a breath-hold at mid expiration) oriented to be perpendicular to the anterior abdominal wall were obtained. Axial gradient-echo T1-weighted MR images was used with a repetition time of 132 ms and an echo time of 4.2 ms, flip-angle of 80° with a 42 cm^2^ field of view and a matrix of 256×256 pixels (in-plane spatial resolution 1.64 mm×1.64 mm). The body coil was used for image acquisition. The total research time was about 20 seconds which was within the breath-hold tolerance of all subjects.

The acquired MRI images were transferred to a computer for digital reconstruction to determine the muscle cross sectional area (CSA). The volume for *iliopsoas* (*iliacus* and *psoas* together) and *gluteal muscles* (*gluteus maximus*, *gluteus medius* and *gluteus minimus* together) were calculated from L1–L2 intervertebral disc to the pubic symphysis. Each image was labeled referred to discal spaces, cranial aspect of coxofemoral joint and pubic symphysis using sagittal and axial scout images. All calculations were carried out by the same investigator, who was blinded to arm dominance, using a specially designed image analysis software (SliceOmatic 4.3, Tomovision Inc., Montreal, Canada), as described elsewhere [23]. A threshold was selected for adipose and lean tissues on the basis of the grey-level image pixel histograms to identify tissue area and the tissue boundaries were manually traced [23].

The total volume (V_total_) of the IL and GL were assessed in each subject [24]. Regional volumes of IL and GL were also calculated for comparative purposes as described elsewhere [4]. Degree of asymmetry was assessed by the calculation of a ratio of the volume of the dominant and non-dominant side [DND = ((non-dominant – dominant volume)×100))/dominant volume].

### Statistical analysis

Results are presented as means ± standard deviation, except for the bar figures which are presented as means ± standard error of the mean. Side-to-side comparisons were carried out using the paired Student's t-test adjusted for multiple comparisons using the Bonferroni-Holm method. Analyses of covariance were performed to compare differences across groups, with age, BMI (body mass index) and total length of *iliopsoas* and *gluteal muscles* as covariates. Between-groups segment-to-segment comparisons were adjusted for the length of segment under scrutiny. The relationship between muscle length and muscle volumes into each group was determined by linear regression analysis. To test the similarity of slopes and intercepts of these relationships, the corresponding t-test was applied for the model: *Y_ij_ = α_i_ + β_i_X_ij_ + ε_ij_* for *i* = 1,2 (1 = soccer players, 2 = controls) and *j* = 1,…, *n_1_* being *ε_ij_* i.i.d. random variables following a distribution N(0, σ_1_). SPSS package (SPSS Inc., Chicago, IL, USA, v15.0) for personal computers was used for the statistical analysis. Significant differences were assumed when P<0.05.

## Results

### Physical characteristics and length of iliopsoas and gluteal muscles

Physical characteristics and total and regional length of *iliopsoas* and *gluteal muscles* are summarized in Table 1. SP, TP and controls were comparable in age, body mass and body mass index. SP and TP were significantly taller than controls (P<0.05). The length of *iliopsoas* and *gluteal muscles* was longer in SP and TP than in CG (P<0.01). *Gluteal muscles* were longer in TP than in SP (P<0.01).

### Differences into each group

#### Muscle volumes

**Figure 1:**
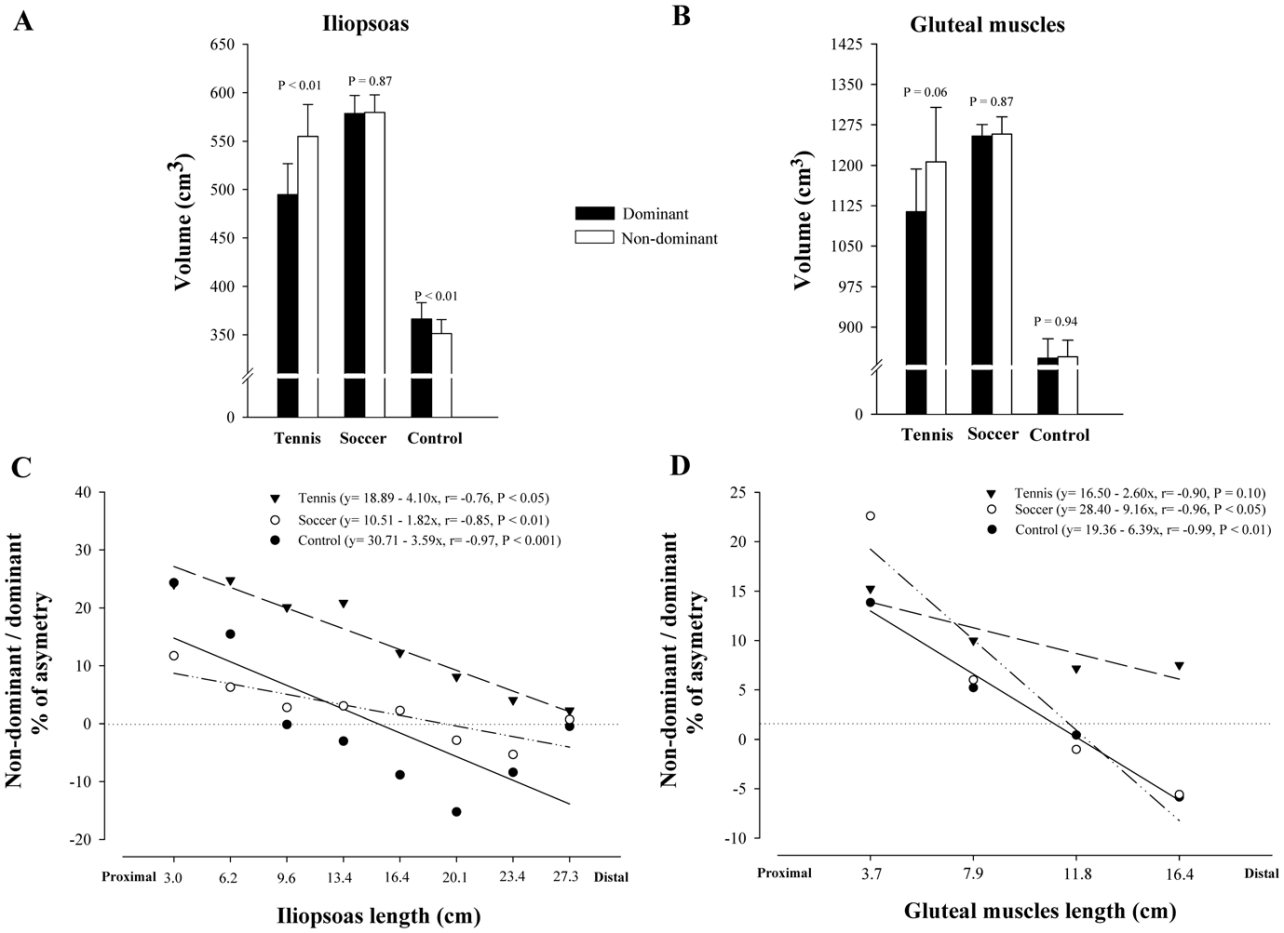
Side-to-side asymmetries in the volume of *iliopsoas* and *gluteal muscles* in tennis players, soccer players and non-athletes. Volume of the dominant and non-dominant *iliopsoas* (A), and *gluteal muscles* (B) into each group. Relationship between the asymmetry in muscle volume of the dominant and non-dominant sides (expressed in percentage), and the *iliopsoas* (C) and *gluteal muscles* (D) segments ordered in the rostro-caudal direction (TP: black triangles; SP: white circles; CG: black circles). The slopes and intercepts were significantly lower in SP than in TP (P<0.01 and P<0.001, respectively) in *iliopsoas*, and in TP than in CG in *gluteal muscles* (P<0.05).

Table 2 summarizes total and regional muscle volumes of *iliopsoas* and *gluteal muscles* in SP, TP and controls. In TP, total volume of the non-dominant IL was 13% greater than the dominant (P<0.01), in CG the dominant side was 4% greater than the contralateral (P<0.01) and in SP both sides had similar volumes (P = 0.87) (Fig. 1A). Tennis players showed a trend to greater volume in the non-dominant compared to the dominant *gluteal muscles* (8%, P = 0.06), whilst similar GL muscles volumes were observed in both sides in SP and CG (P = 0.87 and P = 0.94, respectively) (Fig. 1B).

**Table 2:** *Iliopsoas* and *gluteal muscles* volumes (values expressed in cm^3^, mean ± SD) and asymmetries.

*ILIOPSOAS*																								
	Tennis Players								Soccer Players								Controls							
	Dominant			Non-dominant				ASY (%)	Dominant			Non-dominant				ASY (%)	Dominant			Non-dominant				ASY (%)
**S1**	18.0	±	9.4	20.5	±	8.5	*P = 0.32*	24	27.6	±	9.2	30.1	±	7.5	*P = 0.18*	12	16.8	±	5.5	20.6	±	5.8	*P<0.01*	24
**S2**	37.7	±	11.5	45.3	±	8.7	*P<0.05*	25	46.7	±	11.2	49.0	±	9.1	*P = 0.66*	6	31.6	±	9.9	36.0	±	10.1	*P = 0.12*	16
**S3**	54.7	±	9.7	65.5	±	12.0	*P<0.01*	20	72.0	±	14.8	74.0	±	16.4	*P = 0.57*	3	44.3	±	6.8	44.2	±	7.0	*P = 0.91*	0
**S4**	89.0	±	20.9	106.8	±	22.1	*P<0.001*	21	109.8	±	14.7	112.7	±	13.6	*P = 0.79*	3	60.2	±	12.0	58.6	±	13.2	*P = 0.34*	−3
**S5**	90.7	±	13.5	101.2	±	11.6	*P = 0.15*	12	96.0	±	11.3	98.1	±	11.8	*P = 0.57*	2	70.7	±	9.1	64.2	±	6.2	*P<0.05*	−9
**S6**	95.5	±	20.0	103.0	±	23.6	*P = 0.40*	8	109.0	±	24.0	103.6	±	18.1	*P = 0.75*	−3	70.9	±	12.9	59.4	±	6.0	*P = 0.11*	−15
**S7**	58.6	±	15.5	61.1	±	18.2	*P = 0.68*	4	63.3	±	18.6	58.6	±	14.5	*P = 0.32*	−5	39.2	±	4.5	35.8	±	4.8	*P = 0.28*	−8
**S8**	50.6	±	17.2	51.5	±	16.9	*P = 0.31*	2	53.8	±	13.5	53.4	±	11.8	*P = 0.84*	1	32.5	±	4.6	32.4	±	7.0	*P = 0.96*	−1
**Total**	494.8	±	90.6	555.0	±	92.8	*P<0.01*	13	578.3	±	73.2	579.5	±	70.9	*P = 0.87*	0	366.3	±	41.3	351.2	±	35.7	*P<0.01*	−4
***GLUTEAL MUSCLES***																								
**S1**	74.3	±	27.3	80.6	±	26.3	*P = 0.83*	15	74.5	±	22.0	88.9	±	31.4	*P = 0.17*	23	71.1	±	24.6	78.1	±	24.7	*P = 0.38*	14
**S2**	276.7	±	79.7	301.3	±	83.4	*P = 0.18*	10	329.0	±	48.0	346.8	±	54.2	*P = 0.23*	6	238.3	±	55.2	246.2	±	47.9	*P = 0.66*	5
**S3**	398.4	±	55.1	427.3	±	69.4	*P = 0.13*	7	427.1	±	32.0	422.6	±	35.8	*P = 0.42*	−1	273.8	±	43.9	275.3	±	50.5	*P = 0.87*	0
**S4**	364.4	±	80.5	397.4	±	121.9	*P = 0.24*	8	423.9	±	52.8	399.7	±	60.0	*P = 0.13*	−6	259.0	±	38.3	245.2	±	50.4	*P = 0.20*	−6
**Total**	1113.8	±	225.0	1206.6	±	285.3	*P = 0.06*	8	1254.4	±	81.5	1258.0	±	122.9	*P = 0.87*	0	842.2	±	87.9	844.9	±	75.1	*P = 0.94*	1

ASY: Asymmetry between the dominant and non-dominant sides ((Non-dominant-Dominant)*100)/Dominant.; S1–S8: From segment 1 to segment 8.

Comparisons are made between dominant and non-dominant sides into each group.

An inverse relationship was observed between the length of IL starting from the proximal segment and the degree of asymmetry in muscle volume expressed as the non-dominant/dominant ratio in TP (r = −0.97, P<0.001), SP (r = −0.85, P<0.01) and controls (r = −0.76, P<0.05) (Fig. 1C). The slopes and intercepts were significantly lower in SP than in TP (P<0.01 and P<0.001, respectively). The intercept was significantly higher in TP than in controls (P<0.001), while the slopes were similar in TP and CG (P = 0.74). Not significant differences were observed in the slopes and intercepts between SP and CG (P = 0.16 and P = 0.62, respectively). An inverse relationship was also observed between the length of GL and the degree of asymmetry in muscle volume in SP (r = −0.96, P<0.05) and controls (r = −0.99, P<0.01), TP showed a trend in the same direction (r = −0.90, P = 0.10) (Fig. 1D). The slopes and intercepts were significantly lower in TP than in CG (P<0.05), and similar between SP and CG (P = 0.25 and P = 0.44, for the slopes and intercepts, respectively). When TP and SP were compared, the slope was significantly lower in TP (P<0.05) and the intercepts were similar (P = 0.30).

#### Cross sectional area

Table 3 summarizes the maximum CSA into each segment. In TP, the CSA of *iliopsoas muscle* was greater in the non-dominant than in the dominant side in segments 2–5 (P<0.05). In CG, the CSA of the non-dominant *iliopsoas* was greater than the dominant in segment 1 (P<0.001). Side-to-side differences in the CSA of IL were not statistically significant in any segmental level in SP. Side-to-side differences in *gluteal muscles* were not statistically significant in any segmental level in TP, SP and CG.

**Table 3:** *Iliopsoas* and *gluteal* cross sectional areas (values expressed in cm^2^, mean ± SD) and asymmetries.

*ILIOPSOAS*																								
	Tennis Players								Soccer Players								Controls							
	Dominant			Non-dominant				ASY (%)	Dominant			Non-dominant				ASY (%)	Dominant			Non-dominant				ASY (%)
**S1**	7.5	±	3.4	9.3	±	2.9	*P = 0.08*	31	11.6	±	3.6	12.2	±	2.4	*P = 0.19*	9	7.3	±	1.9	8.8	±	1.9	*P<0.001*	21
**S2**	13.4	±	3.5	16.6	±	2.6	*P<0.01*	27	16.8	±	3.7	17.5	±	3.2	*P = 0.23*	5	11.2	±	2.6	12.9	±	2.7	*P = 0.13*	16
**S3**	18.1	±	3.4	21.0	±	3.5	*P<0.001*	16	22.5	±	3.8	23.1	±	3.9	*P = 0.21*	3	16.4	±	2.0	15.7	±	2.8	*P = 0.29*	−5
**S4**	27.5	±	5.9	31.2	±	5.2	*P<0.05*	15	31.7	±	4.0	32.3	±	3.4	*P = 0.45*	2	20.9	±	3.4	19.6	±	2.8	*P = 0.14*	−6
**S5**	31.9	±	4.0	35.2	±	3.2	*P<0.05*	11	33.4	±	3.8	34.0	±	4.2	*P = 0.25*	2	25.2	±	3.4	22.3	±	2.5	*P = 0.14*	−11
**S6**	29.1	±	4.4	31.7	±	4.0	*P = 0.26*	10	31.7	±	4.6	30.7	±	4.8	*P = 0.31*	−3	23.3	±	2.2	19.7	±	1.9	*P = 0.14*	−15
**S7**	20.6	±	4.3	21.7	±	5.0	*P = 0.38*	6	21.1	±	4.6	19.2	±	3.4	*P = 0.64*	−7	16.0	±	1.9	13.5	±	1.7	*P = 0.16*	−15
**S8**	14.6	±	4.2	15.7	±	4.5	*P = 0.25*	7	15.0	±	3.3	15.0	±	3.2	*P = 0.98*	1	9.3	±	0.9	9.5	±	1.4	*P = 0.78*	3
***GLUTEAL MUSCLES***																								
**S1**	33.1	±	8.7	36.7	±	9.9	*P = 0.14*	13	34.7	±	8.9	39.0	±	11.5	*P = 0.43*	14	34.6	±	10.8	37.4	±	10.0	*P = 0.48*	14
**S2**	84.0	±	16.3	92.7	±	19.5	*P = 0.06*	10	95.2	±	7.8	99.2	±	12.1	*P = 0.26*	5	78.8	±	9.3	80.2	±	5.0	*P = 0.72*	3
**S3**	105.2	±	13.7	112.7	±	19.1	*P = 0.18*	7	110.3	±	7.0	109.7	±	7.9	*P = 0.59*	−1	85.8	±	6.5	85.5	±	8.2	*P = 0.92*	0
**S4**	94.1	±	10.0	102.9	±	23.3	*P = 0.26*	8	99.7	±	11.2	96.2	±	10.8	*P = 0.36*	−3	76.4	±	12.3	73.0	±	15.1	*P = 0.34*	−5

ASY: Asymmetry between the dominant and non-dominant sides ((Non-dominant-Dominant)*100)/Dominant; S1–S8: From segment 1 to segment 8.

Comparisons are made into each group between dominant and non-dominant sides.

### Differences between groups: Tennis vs control

#### Muscle volumes

**Figure 2:**
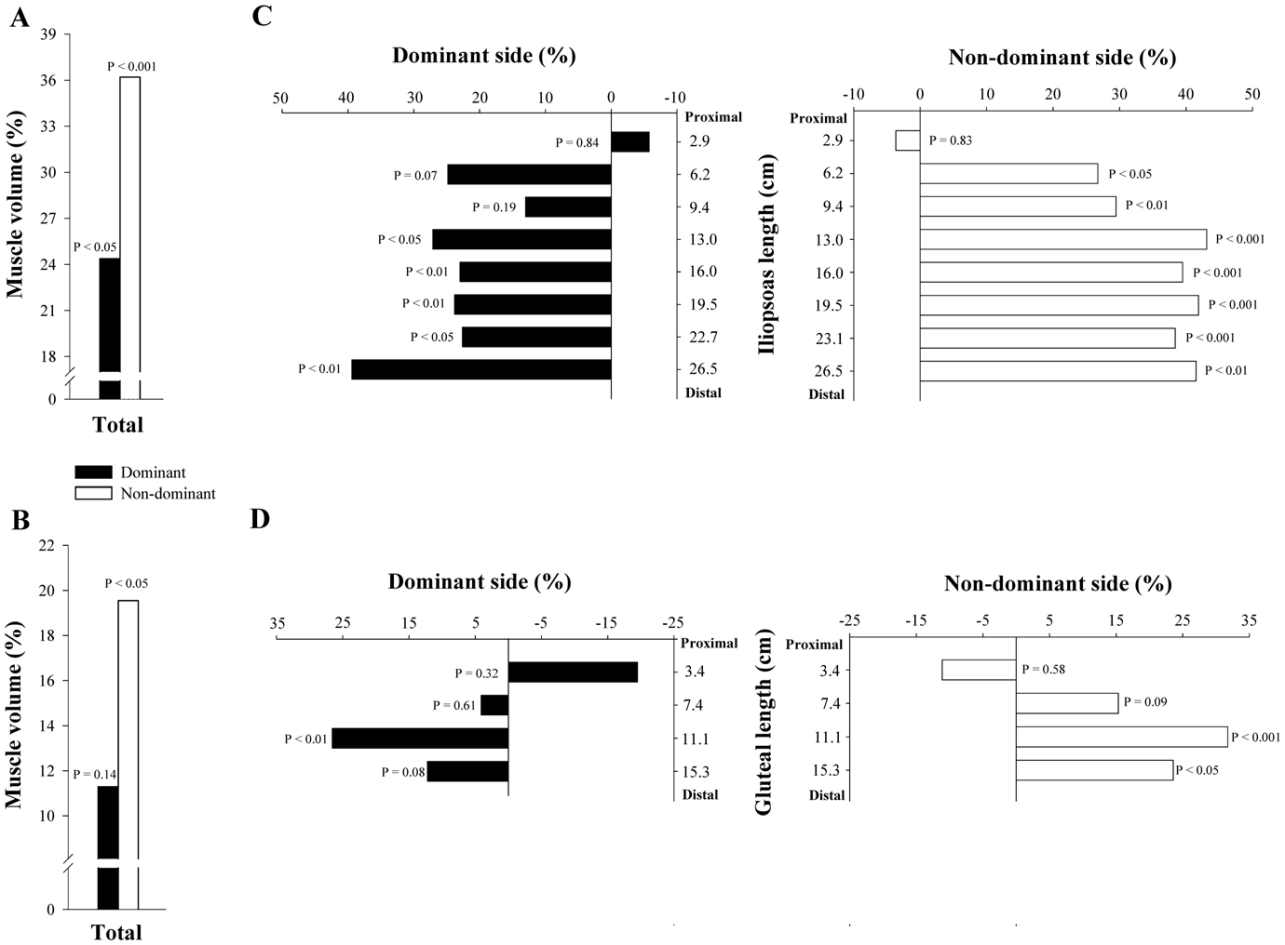
Differences in total volume and CSA (segment by segment), expressed in percentage, between tennis players and non-athletes. Difference in total volume of dominant and non-dominant *iliopsoas* (A) and *gluteal muscles* (B); Difference in CSA, segment by segment, of dominant and non-dominant *iliopsoas* (C) and *gluteal muscles* (D). All comparisons are adjusted for the length of the corresponding muscle, age and BMI.

**Figure 3:**
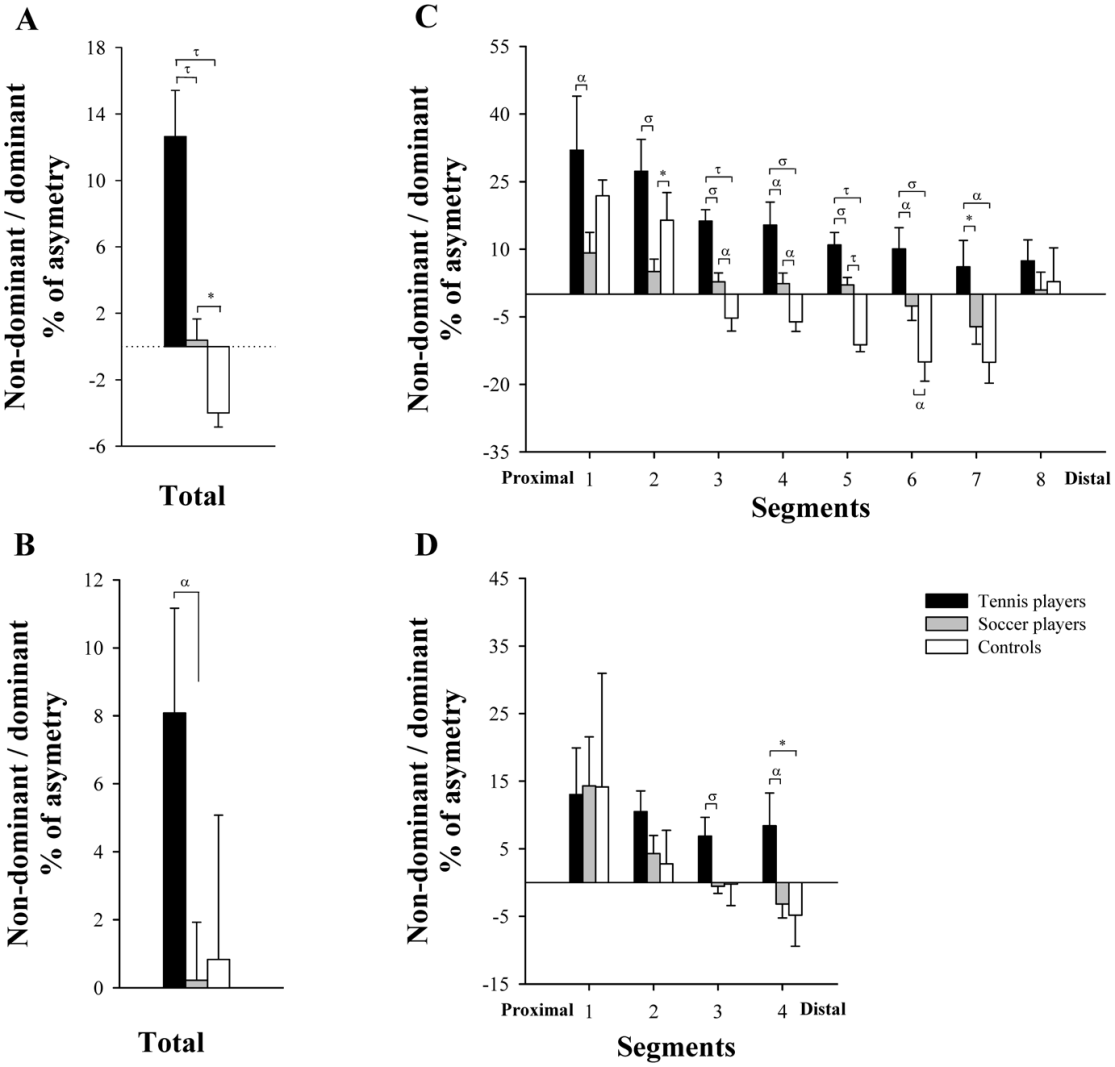
Differences between tennis players, soccer players and non-athletes in the percentage of asymmetry of *iliopsoas* and *gluteal muscles*. Percentage of asymmetry in total volume of *iliopsoas* (A) and *gluteal muscles* (B); Percentage of asymmetry in CSA, segment by segment, of *iliopsoas* (C) and *gluteal muscles* (D). α P<0.05, σ P<0.01, τ P<0.001, * P = 0.06.

Compared to controls, TP had 26% (P<0.01) and 37% (P<0.001) more total IL muscle volume in the dominant and non-dominant sides, respectively. After adjusting for age, the length of *iliopsoas* and BMI as covariates TP had 24% (P<0.05) and 36% (P<0.001) more total volume than controls in the dominant and non-dominant sides, respectively (Fig 2A). The ratio DND of the IL volumes was greater in TP than in controls (12.6±7.9 vs −4.0±2.1%, respectively, P<0.001) (Fig. 3A).

Tennis players had 24% (P<0.05) and 30% (P<0.01) more total volume in the dominant and non-dominant GL than controls. After controlling for age, the length of *gluteal muscles* and BMI as covariates, TP had 20% greater muscle volume in the non-dominant side (P<0.05) than controls, whilst no significant differences were observed between TP and CG in the volume of the dominant side (11% greater in TP, P = 0.14) (Fig 2B). The ratio DND of the GL volumes was similar in TP and in controls (8.1±8.7 vs 0.8±10.4%, respectively, P = 0.18) (Fig. 3B).

#### Cross sectional areas

Compared to controls, the CSA of the *iliopsoas muscle* of TP were greater in segments 4–8 of the dominant and non-dominant sides (P<0.01). After controlling for age, the length of each *iliopsoas* segment and BMI as covariates TP had higher CSA than controls in segments 4–8 of the dominant side and 2–8 of the non-dominant side (P<0.05) (Fig. 2C). A positive correlation was observed between muscle length starting from the inter-discal L1–L2 space and the mean difference in CSA between TP and CG in the dominant (r = 0.75, P<0.05) and the non-dominant side (r = 0.76, P<0.05), adjusted for age, the length of each segment and BMI. Between group differences in the degree of asymmetry of *iliopsoas muscle* were greater in TP than in controls in segment 3–7 (P<0.05) (Fig. 3C).

Compared to controls, the CSA of *gluteal muscles* of TP were greater in segments 3 and 4 of the dominant and non-dominant sides (P<0.05). After controlling for age, the length of each *iliopsoas* segment and BMI as covariates TP had higher CSA than controls in segment 3 of the dominant side (P<0.01) and segments 3 and 4 of the non-dominant side (P<0.05) (Fig. 2D). Between group differences in the degree of asymmetry of *gluteal muscles* were similar in TP and in CG in all segmental levels, although TP showed a trend of greater asymmetry in the more distal segment (P = 0.07) (Fig. 3D).

### Differences between groups: Soccer vs control

#### Muscle volumes

**Figure 4:**
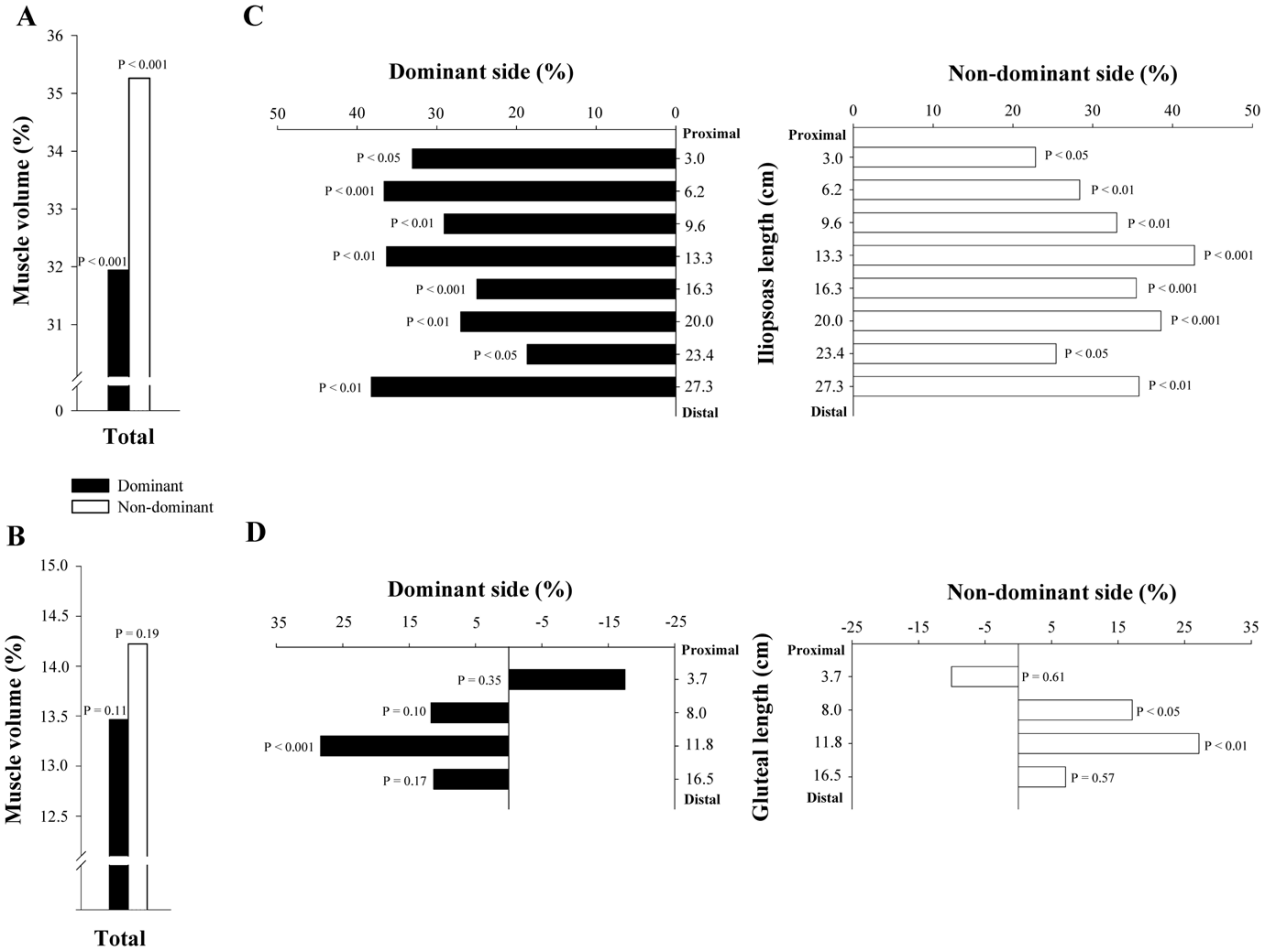
Differences in total volume and CSA (segment by segment), expressed in percentage, between soccer players and non-athletes. Difference in total volume of dominant and non-dominant *iliopsoas* (A) and *gluteal muscles* (B); Difference in CSA, segment by segment, of dominant and non-dominant *iliopsoas* (C) and *gluteal muscles* (D). All comparisons are adjusted for the length of the corresponding muscle, age and BMI.

Soccer players had 37% and 39% more total muscle volume in the dominant and non-dominant sides of IL compared to controls, respectively (P<0.001). After controlling for age, the length of *iliopsoas* and BMI as covariates soccer players had 32% and 35% more total muscle volume in the dominant and non-dominant sides compared to controls, respectively (P<0.001) (Fig. 4A). The ratio DND of the IL volumes tended to be greater in soccer players than in controls (0.4±5.0 vs −4.0±2.1%, respectively, P = 0.06) (Fig. 3A).

Soccer players had 33% more total muscle volume in the dominant and non-dominant GL than controls (P<0.001). After controlling for age, the length of *gluteal muscles* and BMI as covariates soccer players had 13% (P = 0.11) and 14% (P = 0.19) greater volumes than controls in the dominant and non-dominant sides, respectively, but these differences were not statistically significant (Fig. 4B). The ratio DND of the GL volumes was similar in soccer players and in controls (0.2±6.6 vs 0.8±10.4%, respectively, P = 0.87) (Fig. 3B).

#### Cross sectional areas

The CSA of the *iliopsoas muscle* of SP was greater than in controls in all segmental levels of the dominant and non-dominant sides (P<0.01). This difference remained statistically significant after adjusting for age, the length of each *iliopsoas* segment and BMI as covariates (P<0.05) (Fig. 4C). Excluding segment 8, an inverse correlation was observed between muscle length starting from the inter-discal L1–L2 space and the mean difference in CSA between SP and CG in the dominant IL (r = 0.79, P<0.05), adjusted for age, the length of each segment and BMI. Between group differences in the degree of asymmetry of *iliopsoas muscle* was greater in SP than in controls in segment 3–6 (P<0.05) (Fig. 3C).

In SP, the CSA of *gluteal muscles* was greater than in controls in segments 2–4 of the dominant and non dominant sides (P<0.01). After controlling for age, the length of each *iliopsoas* segment and BMI as covariates, SP had higher CSA than controls in segment 3 of the dominant side (P<0.001) and segments 2 and 3 of the non-dominant side (P<0.05) (Fig. 4D). Between group differences in the degree of asymmetry of GL was similar in both groups in all segmental levels (Fig. 3D).

### Differences between groups: Tennis vs. soccer

#### Muscle volumes

Soccer players had 17% more muscle volume in the dominant *iliopsoas muscle* than TP (P<0.05), whilst a similar muscle volume was observed in the non-dominant side (4%, P = 0.48). After controlling for age, the length of *iliopsoas* and BMI as covariates SP showed a trend to greater muscle volumes in the dominant side than TP (10%, P = 0.08), whilst the non-dominant side was similar in both groups (1%, P = 0.78). The ratio DND of the IL volumes was greater in TP than in SP (12.6±7.9 vs 0.4±4.6%, respectively, P<0.001) (Fig. 3A).

The muscle volume of the dominant side of *gluteal muscles* was 12% greater in SP than in TP (P<0.05), whilst the non-dominant side was similar in both groups (4%, P = 0.64). After controlling for age, the length of *gluteal muscles* and BMI as covariates, the muscle volume of the dominant and non-dominant sides was similar in SP and in TP (3%, P = 0.61 and 6%, P = 0.31, respectively). The ratio DND of the GL volumes was greater TP than in SP (8.1±8.7 vs 0.2±6.6%, respectively, P<0.05) (Fig. 3B).

#### Cross sectional areas

Compared to TP, the CSA of the *iliopsoas muscle* of SP was greater in segments 1–3 of the dominant side (P<0.01) and in segment 1 of the non-dominant side (P<0.05). After adjusting for age, the length of each *iliopsoas* segment and BMI as covariates SP had higher CSA than TP in segments 1 and 2 of the dominant side (P<0.05), and also in segments 1 and 7 of the non-dominant side (P<0.05). Between group differences in the degree of asymmetry of *iliopsoas muscle* was greater in TP than in SP in segments 1–7 (P<0.05) (Fig. 3C).

The CSA of *gluteal muscles* was similar in SP and TP in all segmental levels of the dominant and non-dominant sides. After controlling for age, the length of each *iliopsoas* segment and BMI as covariates TP had higher CSA than SP in segment 4 of the non-dominant side (P<0.05). Between group differences in the degree of asymmetry of GL was greater in TP than in SP in segment 3 and 4 (P<0.05) (Fig. 3D).

## Discussion

A unique finding of the study was to determine the volume and degree of asymmetry of *iliopsoas* and *gluteal muscles* in professional male tennis and soccer players. The dominant and non-dominant *iliopsoas muscles* were hypertrophied in tennis (24 and 36%, respectively) and soccer players (32 and 35%, respectively), compared to non-athletes controls. Both sports modified the side-to-side asymmetry of *iliopsoas muscle* observed in control group, who had 4% more volume in the dominant side (the side of the dominant arm). Tennis reversed the asymmetry observed in non-active controls (the non-dominant *iliopsoas* was 13% greater than the dominant), whilst soccer compensated it. Our study also shows that the magnitude of asymmetry of *iliopsoas* decreased progressively from proximal to distal regions in TP, SP and CG. The slope of this relationship was lower in soccer players due to a greater hypertrophy of the dominant *iliopsoas* in the proximal segments. On the other hand, the present study shows that soccer does not induce a significant increase in the muscle volume of *gluteal muscles* compared to controls, whilst in TP the hypertrophy is asymmetric, the non-dominant side is 20% greater and the dominant side is similar to controls.

The present study shows that the magnitude of hypertrophy of *iliopsoas muscle* was similar in TP and in SP (30 and 33%, respectively, both sides considered together). The hypertrophy of IL reflects the large loads sustained by this muscle in both sports. We cannot compare our results with other studies analyzing the volume of *iliopsoas muscle*, but the degree of hypertrophy of *iliopsoas* can be considered high if one takes into consideration the hypertrophy of other muscle groups into the same sports [4], [19]. It has been previously reported that compared to non-athletes, tennis players increase the volume of the muscles of the dominant arm a mean 27% [19], whilst the *rectus abdominis* is hypertrophied a mean 58% in tennis [4] and 26% in soccer players [25]. Interestingly, tennis was associated to 35% greater volume in the non-dominant *rectus abdominis* than in the contralateral side [4], whilst in soccer players both sides had similar volumes [25]. Our study shows that tennis and soccer induce a similar effect on *iliopsoas muscle* and *rectus abdominis*. In TP, the non-dominant *iliopsoas* was 13% greater than the dominant and SP had similar volumes in both sides.

The asymmetric hypertrophy of *iliopsoas* induced by tennis reversed the side-to-side asymmetry observed in control subjects, who had 4% more volume in the dominant side. The shift in the side-to-side relationship observed in tennis players can only be explained by the asymmetric nature of tennis strokes. Studies using cinematography have shown that trunk flexion and rotation are main contributors of power generation in the service and forehand strokes [26]. During the backswing the shoulders rotates more than the hip (storage of elastic energy) to allow a more vigorous trunk flexion and rotation in the early stages of the forwardswing [26]. This forward rotation requires a solid foundation in the non-dominant side for the torques generated by the dominant arm. The higher level of hypertrophy of non-dominant compared to dominant IL sustains this hypothesis.

Similar arguments could explain the effects of kicking on the hypertrophy *iliopsoas* in soccer players. The present study shows that soccer compensated the side-to-side asymmetry observed in non-athletes (4%) due to a greater hypertrophy of the non-dominant than the dominant IL (3%, NS). Soccer induced a similar degree of hypertrophy of the non-dominant IL than tennis (35 and 36%, respectively), but the hypertrophy of the dominant IL was 10% greater in soccer than in tennis players (34 and 24%, respectively, P = 0.08). The larger hypertrophy of the dominant IL in soccer players could be attributed to the active participation of this muscle during kicking [12], or less likely by a greater compensatory effect of sprinting in soccer. Most soccer players have a favored foot for kicking. The dominant IL is the most active muscle of the dominant leg during the entire kicking motion [12]. But *iliopsoas* is also very active during sprinting [1] and sprinting constitutes one of the most important activities in soccer [27], and not in tennis [28]–[31]. Soccer demands longer sprints than tennis to obtain an advantageous position to receive the ball or to defend a player [32], whereas tennis is more characterized by quick movements in varied directions with shorter displacements (mean 3 m per shot) [29]. Future studies should analyze the effects of soccer on IL muscle by playing positions, as this aspect determines the distance and intensity of sprinting in soccer [32] and could influence the degree of asymmetry of *iliopsoas*.

The magnitude of asymmetry between *iliopsoas muscles* decreased progressively from proximal to distal regions in SP, TP and controls. As illustrated in figure 1C, the non-dominant IL was greater than the dominant in the proximal regions but these differences were progressively reduced in regions closer to pubic symphysis in soccer and in tennis players. Similarly, in non-athletes the non-dominant IL was greater than the dominant in the proximal segments (1–3), but dominant IL became larger than the non-dominant from segments 3 to 8. Interestingly, the slope of the relationship was significantly smaller in soccer than in tennis players. The reason was that contrary to tennis players and controls, the dominant IL of soccer players was more hypertrophied in proximal than in distal regions, with the exception of segment 8 (see figure 4A). In support, studies conducted in elite Australian Rules Football (AFL) consistently showed that the dominant *psoas major* muscle was significantly larger than the non-dominant [33], [34]. But in our soccer players we found no significant side-to-side differences in any segmental level of *iliopsoas muscle.* This difference might be attributed to a higher demand of the dominant *psoas major* in AFL players than in soccer players, probably due to the greater amplitude of most kicking actions.

In tennis players the degree of hypertrophy of the dominant and non-dominant IL increased progressively from proximal to distal regions (see figure 2C), with the non-dominant IL being significantly greater than the dominant in the proximal segments (15 cm from L1/L2 in rostro caudal direction). Side-to-side differences in *iliopsoas muscle* had not been previously studied in tennis players. Our results show important differences from cricket fast bowlers who had similar CSA in both sides of *psoas major* muscle (measured at L3/L4 discal level) [35]. This suggest that the asymmetric hypertrophy of *iliopsoas muscle* in tennis players might be influenced not only by the tennis serve, which is a similar movement than bowling in cricket, but also by the forehand stroke [26].

The present study shows that the hypertrophy of *gluteal muscles* in tennis players is asymmetric. In concordance with our results in non-active controls, recent studies have reported similar volumes in both sides of *gluteus maximus* [36], *medius* and *minimus* [2] in healthy non-active population. Compared to non-active controls the non-dominant side of the tennis players was hypertrophied a mean 20%, whilst no between-group differences were observed in the dominant side (11% greater volume in TP, NS). It is well documented that the lower limb drive, together with trunk rotation, is a key factor to increase racket speed at impact during tennis strokes [26], [37], [38]. To increase power during tennis strokes, the players commences with a flexion of the lower limbs so that the body can be moved towards the court. The greater hypertrophy of the non-dominant GL indicates that this muscle contributes to increase the force generating capacity and peak power during tennis strokes more than the dominant GL [39], [40].

Based on electromiographic studies we hypothesized that soccer would also induce the asymmetric development of *gluteal muscles*, with greater volume in the dominant compared to the non-dominant side, reflecting greater stretch-shortening loads of the dominant leg during kicking [12]. Our results show that soccer players had similar total volumes in both *gluteal muscles*, and similar total volumes compared to non-active controls (14% more volume in SP than in CG in both sides, NS). Therefore, in terms of total volume soccer does not induce the hypertrophy of *gluteal muscles*. But the segmental analysis showed important between group differences. The CSA of non-dominant segments 2 and 3, and dominant segment 3 was greater in SP than in controls, whilst tennis players had greater CSA than controls in segments 3 and 4 of the non-dominant and also in segment 3 of the dominant side (see figure 2D and 4D). Therefore, a differential degree of hypertrophy in the upper and lower segments of *gluteal muscles* is developed depending on the sports requirements. In support, Grimaldy et al. [36] observed that the magnitud of hypertrophy of the upper and lower *gluteus maximus* was different in subjects with osteoarthritis. It remains to be determined the effects of soccer and tennis on the total and regional muscle volume of *gluteus maximus*, *medius* or *minimus* independently to identify specific adaptations into these muscles.

Common injuries in tennis and soccer players have been associated to side-to-side asymmetries in *iliopsoas* and *gluteal muscles* [5], [6], [11], [22], [41]. The asymmetric hypertrophy of iliopsoas *muscle* can lead to lower back pain [5], chronic groin pain [6], *iliopsoas* bursitis and tendinitis [41] or greater trochanteric pain syndrome [11] in these sports. The present study gives useful information for a better knowledge of the influence of the asymmetric hypertrophy of IL and GL muscles on these injuries. For example, the greater hypertrophy of the distal part of *iliopsoas muscle* observed in our soccer players could predispose to develop *iliopsoas* bursitis and tendinitis [41], [42]. An unique finding of the present study was that tennis induces an asymmetric hypertrophy of *iliopsoas* and *gluteal muscles* and soccer compensates the asymmetry observed in non-active controls. It remains to be determined whether these different patterns can modify the risk of lower back pain and chronic groin pain [6], [13], [14], [43]. Moreover, the low magnitude of hypertrophy of *gluteal muscles* observed in our soccer players could be associated to a greater risk of anterior cruciate ligament injuries [22], [44].

In summary, the present study describes for the first time the effects of professional soccer and tennis on the volume of the *iliopsoas* and *gluteal muscles*. Our study indicates that both sports are associated with a similar increase in the muscle volume of *iliopsoas* (30 and 33% for tennis and soccer, respectively, both sides considered together). However, the hypertrophy of *iliopsoas* is asymmetric in tennis players (the non-dominant *iliopsoas* was 13% greater than the dominant), whilst soccer players had similar volumes in both sides. Our study also shows that the magnitude of asymmetry of *iliopsoas* decreased progressively from proximal to distal regions in TP, SP and CG. In addition, we have shown that soccer does not induce a significant increase in the muscle volume of *gluteal muscles* compared to controls, whilst in TP the hypertrophy of *gluteal muscles* is asymmetric. It remains to be determined whether the different pattern of hypertrophy of *iliopsoas* and *gluteal muscles* induced by tennis and soccer modifies the risk of injury. These results may be of great importance for coaches and clinicians to design more specific strength training and injury prevention programs.
